# Systematic review of psychological, emotional and behavioural impacts of surgical incidents on operating theatre staff

**DOI:** 10.1002/bjs5.21

**Published:** 2017-10-26

**Authors:** N. Serou, L. Sahota, A. K. Husband, S. P. Forrest, K. Moorthy, C. Vincent, R. D. Slight, S. P. Slight

**Affiliations:** ^1^ School of Pharmacy, Faculty of Medical Sciences Newcastle University Newcastle upon Tyne UK; ^2^ Cardiothoracic Department, Freeman Hospital Newcastle upon Tyne Hospitals NHS Foundation Trust Newcastle upon Tyne UK; ^3^ Pharmacy Department, Freeman Hospital Newcastle upon Tyne Hospitals NHS Foundation Trust Newcastle upon Tyne UK; ^4^ Theatres and Anaesthetics, Surgery, Cancer and Cardiovascular Division Imperial College Healthcare NHS Trust London UK; ^5^ Perioperative Practice and Operating Department Practice, College of Nursing, Midwifery and Healthcare University of West London London UK; ^6^ Department of Experimental Psychology University of Oxford Oxford UK; ^7^ Center for Patient Safety Research and Practice, Division of General Internal Medicine Brigham and Women's Hospital and Harvard Medical School Boston Massachusetts USA

## Abstract

**Background:**

Adverse surgical incidents affect both patients and health professionals. This study sought to explore the effect of surgical incidents on operating theatre staff and their subsequent behaviours.

**Methods:**

Eligible studies were primary research or reviews that focused on the effect of incidents on operating theatre staff in primary, secondary or tertiary care settings. MEDLINE, Embase, CINALH and PsycINFO were searched. A data extraction form was used to capture pertinent information from included studies and the Critical Appraisal Skills Programme (CASP) tool to appraise their quality. PRISMA‐P reporting guidelines were followed and the review is registered with PROSPERO.

**Results:**

A total of 3918 articles were identified, with 667 duplicates removed and 3230 excluded at the title, abstract and full‐text stages. Of 21 included articles, eight focused on the impact of surgical incidents on surgeons and anaesthetists. Only two involved theatre nurses and theatre technicians. Five key themes emerged: the emotional impact on health professionals, organization culture and support, individual coping strategies, learning from surgical complications and recommended changes to practice.

**Conclusion:**

Health professionals suffered emotional distress and often changed their behaviour following a surgical incident. Both organizations and individual clinicians can do a great deal to support staff in the aftermath of serious incidents.

## Introduction

Medical errors affect up to 16 per cent of patients admitted to hospital^1^ ^2^, with 50 per cent of these occurring when surgical or invasive procedures are performed^2^. Common examples include wrong site surgery, retained foreign objects, and insertion of the wrong implant or prosthesis^3^. Patients and health professionals are both affected, the latter group being recognized as secondary victims of medical error^4^ ^5^. A secondary victim has been defined as ‘a health care provider involved in an unanticipated adverse patient event, medical error and/or a patient related‐injury who becomes victimised in the sense that the provider is traumatised by the event’^5^ ^6^.

Surgical incidents are those events that occur during a surgical or invasive procedure in an operating theatre. They may or may not result in patient harm (near misses, serious adverse events), but still affect the health professionals involved. Surgeons have reported both emotional distress and depression^5^ ^7^, ^8^. Emotional distress symptoms have been likened to those of post‐traumatic stress disorder^9^. Operating theatre nurses and allied health professions involved can also suffer loss of self‐confidence and job dissatisfaction^10^. In the UK, the Care Quality Commission recommended that organizational support be offered to staff at this time and stressed the importance of actively supporting the health and well‐being of staff^11^ ^12^.

Most qualitative studies have focused on the impact of errors occurring outside the operating theatre^4^ ^8^, ^13^, ^14^, ^15^, ^16^, ^17^, ^18^, ^19^, ^20^, or have concentrated on the supporting systems in place for secondary victims after medical errors have occurred^21^. This systematic review was conducted to ascertain the psychological, emotional and behavioural impacts of surgical incidents on operating theatre staff, and how their attitudes might change subsequently. The review considered professional and personal impacts of a surgical incident on operating theatre staff, the safety concerns raised by staff affected by the incident, and the support offered to staff following such a surgical incident.

## Methods

This review followed the Preferred Reporting Items for Systematic Review and Meta‐Analysis Protocols (PRISMA‐P) reporting guidelines^22^. The review was registered with the PROSPERO database (number 420112042415).

### Inclusion and exclusion criteria

Studies were eligible for inclusion if they were primary research or reviews focused on the effect of surgical incidents on operating theatre staff (medical and non‐medical) in primary, secondary and tertiary care settings. A surgical incident was defined as an incident that occurred while performing a surgical or invasive procedure in an operating theatre (including operating room and anaesthetic room) or suite (for example primary care medical centre) that may or may not have resulted in patient harm (near misses, serious incidents and never events). Operating theatre staff were defined as health professionals working in an operating theatre or suite (both medical and non‐medical) covering any specialty and level of expertise.

Articles of interest included data concerning one or more of the following: professional and personal impact of a surgical incident on operating theatre staff, including psychological or emotional consequences that affected staff performance, practices and responses; safety concerns raised by staff affected by a surgical incident; and support offered to staff by their colleagues, seniors, department or organization following a surgical incident. Studies that investigated the impact on patients, malpractice litigation, publications in languages other than English, those related to dentistry, and studies of the impact of other kinds of error not involving invasive procedures were excluded.

### Search strategy and study selection

**Figure 1:**
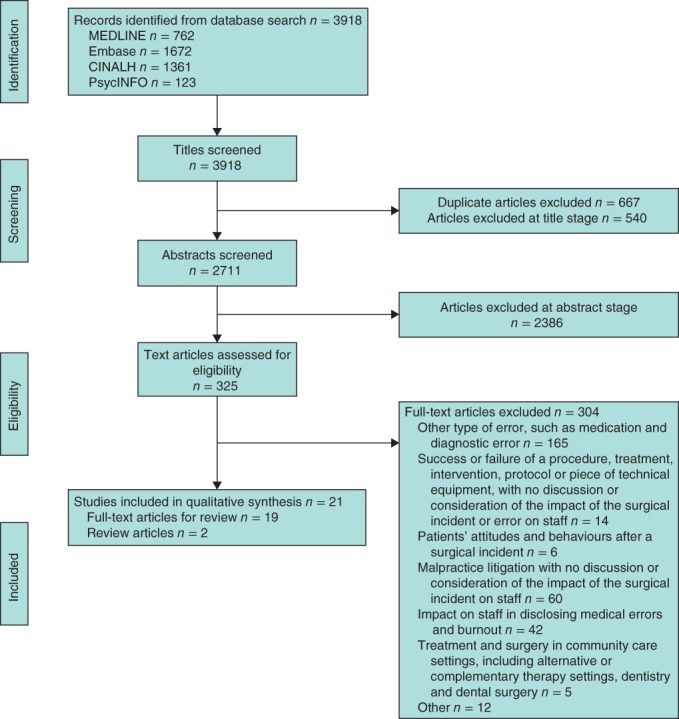
PRISMA diagram showing selection of articles for systematic review

A comprehensive set of search terms were developed based on the definitions of surgical incidents and operating theatre staff. A list of MeSH (medical subject headings) terms and text words were generated; these are provided in *Appendix S1* (supporting information). The following electronic databases were searched in June 2016, from the date of their commencement: MEDLINE in Process (Ovid), Embase (Ovid), the Cumulative Index to Nursing and Allied Health Literature (CINAHL) and PsycINFO. Grey literature was also searched for sources including reports from UK government agencies such as National Patient Safety Agency, and local and regional clinical commissioning groups. Doctoral dissertations, conference proceedings, posters and publications from patient safety conferences, Association for Perioperative Practice (https://www.afpp.org.uk/) and Open Grey (http://www.opengrey.eu) databases were also searched. Studies identified as potentially relevant for inclusion were assessed by two independent reviewers, with arbitration by a third reviewer, if necessary. This involved reviewing all titles, abstracts and full texts, and documenting the reason why each full‐text article was excluded, as outlined in *Fig*. 1.

### Data extraction and synthesis

A customized data extraction form was developed and used to capture pertinent information from included studies. Authors' names, year of study, country where the research was conducted, research methods, types of error discussed, aims and objectives of the research, any recommendations or key findings, and quality assessment of each article were recorded as detailed in *Table S1* (supporting information). Study authors were contacted by e‐mail if further information or clarification was required. A narrative synthesis of the data was undertaken^23^. Emerging and recurrent subthemes relating to the research aims were identified from the included qualitative studies^24^. Quantitative data from the reviewed articles were transformed to a summary of study results and analysed for recurrent patterns across other qualitative studies and review articles included. Details of the initial subthemes and overarching themes thereby extracted are shown in *Table S2* (supporting information).

### Risk of bias (quality) assessment

The quality of included studies was appraised using the Critical Appraisal Skills Programme (CASP) tool^25^. This tool consists of a list of questions, with 1 point awarded for each question up to a maximum score of 10. Quality appraisal of each article was carried out independently by two reviewers. Any disagreements were resolved by discussion with a third additional reviewer. The scores and quality of the selected articles were recorded in the final column of *Table S1* (supporting information).

## Results

A total of 3918 articles were identified. After removal of 667 duplicates, a further 3230 articles were excluded at title (540), abstract (2386) and full‐text (304) stages, leaving a total of 21 articles (19 full‐text and two review articles) (*Fig*. 1). Most of the individual studies selected were conducted in North America (12) followed by western mainland Europe (3), the UK (3) and Australia (1). Of these, 11 studies used quantitative methods, seven qualitative methods, and one mixed methods. The number of individuals who participated in the included qualitative studies ranged from 11 to 31^4^ ^7^, ^15^ ^19^, ^26^, ^27^, ^28^, ^29^. The included studies were assessed for the use of methodological triangulation (use of 2 or more methods), which has been advocated^30^ as a way of safeguarding the ‘validity’ of qualitative studies. As part of the quality assessment of articles, more than half of the selected articles used only one method to obtain the data. A score of 8 out of 10 was deemed to represent a ‘good quality’ paper.

The narrative synthesis of the data from these articles resulted in the recognition of five overarching themes: emotional impact on health professionals, organization culture and support, individual coping strategies, learning from surgical complications, and recommended changes to practice. Of the 19 individual articles, eight had subthemes that were included in the overarching themes. Subthemes from the two selected review articles were included in the overarching themes.

### Emotional impact on health professionals

Health professionals experienced a range of emotions, either immediately or soon after a surgical incident. One of the neurosurgery residents in a Canadian study described this range of emotions as follows: ‘The first thing is probably a bit of shock, and horror. That's quickly replaced by some sort of sadness and depression to some extent … this patient trusted me and my team to do something and we betrayed that trust. So I think for me that's the path of emotions that I follow: initial shock and horror followed by sadness and depression followed by a component of guilt and then self‐doubt’^27^. Other surgeons felt distraught and described how it impacted their ability to perform more mundane tasks: ‘I honestly think I almost crashed into four parked cars before I got out of the parking garage that day. I was so distraught …’^28^. Another surgeon reported having difficulty sleeping, repeatedly recalling the event: ‘I couldn't sleep without thinking about it … I grieve for how badly it makes me feel. I'm always saying I've got to get out of this business because it's hard. It's depressing …’^28^. Some also considered a change in specialty or even early retirement, as they felt unable to cope with another similar incident in the future^28^.

A number of different factors appeared to influence individual reactions to an incident: the individual's resilience and character, their standing within the team hierarchy and the patient outcome. Taking each of these factors in turn, one surgeon explained how some people appear to be unaffected by the event ‘…[*like*] water off a duck's back’^7^, ‘absolute rocks …’^28^, whereas others ‘completely fall to pieces’^7^. Some surgeons agonized over the incident, blaming themselves for their ‘particular lapse’ or how they ‘personally missed something’^7^. These events appeared to ‘live’ with them: ‘… [*I remember*] all their names, I remember their faces, I remember their families’^28^. Another vascular surgical trainee anticipated the impact would be enduring: ‘I'm sure in 20 years’ time I'll still be able to remember this case and what it taught me …'^7^. Junior surgeons appeared to experience more extreme emotions than their senior colleagues^5^ ^7^, ^8^ ^15^, ^25^, ^26^, ^27^, feeling insecure, isolated, and concerned about their reputation and what others might think: ‘… is this an error that I've made that's unforgivable and is it going to affect people's professional opinion of me …’^7^. Some felt that senior colleagues had tried to place the blame on them: ‘he basically pinned the whole thing on us … I don't like it when people finger point and that happens a lot…’^15^.

The cumulative impact of events on practice and emotional experience over time was also highlighted. Surgical incidents that resulted in a patient's death or permanent disability appeared to have more of an emotional impact on surgeons. One vascular surgeon explained ‘… repairing someone's aneurysm, giving them a stroke and then rendering them paraplegic, it would be a terrible outcome … Death, limb loss, paralysis, they're huge and probably affect the impact of complication on your emotions…’^7^. Surgical incidents that occurred during elective procedures also appeared to have more of an impact on operating staff than those that occurred during emergency surgery, perhaps because they considered an unanticipated event less likely^7^ ^15^, ^19^ ^27^.

### Individual coping strategies

Health professionals used different coping strategies in the aftermath of a surgical incident: seeking peer support or counselling, openly discussing the incident with patients and families, reflecting on the incident privately, and implementing changes to their practice. Health professionals often sought the support of their peers or, in some cases, independent counsellors following a surgical incident^8^ ^16^, ^17^ ^27^, ^28^ ^31^. Most surgeons and anaesthetists discussed the event with senior colleagues within their own specialty: ‘the best ‘counselling’ is by talking to a skilled trusted senior anaesthesiologist to put the case into perspective as they can much more understand the context and situation than a counsellor’^31^. In situations where peer support was unavailable, operating theatre staff tended to seek counselling from professional counsellors, with mixed results^31^. Some surgical trainees felt that sharing experiences with their peers helped eliminate self‐doubt and minimize guilt^8^ ^15^, ^27^ ^32^. Simply asking the question: ‘Has this ever happened to you?’ gave one resident reassurance that others had or were ‘going through the same thing’^15^.

Some health professionals chose to discuss the incident with both the patient and their family, which they found helpful^15^. Others chose to reflect privately on the incident, with one vascular registrar explaining how they mentally ‘deconstruct[*ed*] it and replay[*ed*] it’ to assess their degree of responsibility^7^. One surgeon in a Canadian study found it helpful to write a standard operating procedure (SOP) to help prevent such an incident occurring in the future: ‘I will put in [*a SOP*] if I think maybe this piece wasn't right *…* how will I deal with that one next time, maybe that's my coping mechanism’^28^.

### Organization culture and support

The majority of operating theatre staff felt that they received inadequate support from their managers and peers within the organization following a surgical incident. One UK surgeon felt very strongly about the lack of support offered in his hospital: ‘… I don't think the institutions have any knowledge of the difficulties that their consultants face and to my knowledge there are no mechanisms for support, at all. If Surgeon mucks up the Trust's response is to suspend them’^7^. Consequently, operating staff felt reluctant to disclose or discuss any incidents for fear of the consequences. One general surgery trainee explained: ‘If you feel that you're working in a blame environment … you wouldn't be performing to your optimal anyway because you're watching your back the whole time … You might feel that you want to keep things to yourself …’^7^. Junior surgeons often felt reluctant to seek emotional support when they were involved in a surgical incident, as it was seen as a personal weakness^15^ ^27^, ^28^ ^33^.

Different suggestions were proposed to encourage informal and constructive discussions about surgical incidents, including the arrangement of ‘morbidity and mortality’ or ‘deaths and complications’ meetings^7^ ^9^, ^15^ ^28^, ^29^. Designed to encourage professional learning and create a positive patient safety culture, a US trainee found these meetings very supportive and conducive to learning: ‘… I've asked, ‘God, this patient is not doing so well do you think it's because of …?’ … And you know I just try to get education from other people’^15^. In contrast, a UK general surgery trainee recounted a very different experience, with surgeons becoming very defensive at these meetings: ‘… everybody in that room is very defensive and aggressively pursues an angle that puts them in the best possible light and professional rivalries exist …’^7^.

### Learning from surgical complications

Although most studies concentrated on the emotional impact of being involved in a surgical incident, others discussed the importance of personal and organizational learning from such incidents. Sharing the lessons learned was seen as vital for improving patient care^7^ ^13^, ^15^ ^19^, ^26^, ^27^, ^28^ ^33^, ^34^. As outlined above, reflection played an important role in surgical trainees' learning. One surgical trainee in the USA highlighted how important it was to acknowledge mistakes and find ways of preventing them from happening again^15^. In the UK, a general surgeon reflected on how he and his colleagues were less likely to perform the same type of surgery in the future and admitted that this might not be in the best interest of patients: ‘… Well it might make me much less prone to taking any form of risk … and sometimes that's not necessarily in the best interest of the patient …’^7^. Some senior surgeons deconstructed the events that led to an incident and evaluated whether there were any gaps in their knowledge and skills: ‘Is there any knowledge that we don't have and that could have been useful in this case?’^19^. Some studies recommended that meetings to discuss deaths and complications needed to be more structured and blame‐free to encourage open discussions and promote a culture of shared learning within organizations. A US trainee felt that death and complications conferences helped facilitate this learning: ‘This has been a tradition among surgical education for a long time that you present things when they go wrong … I think it is very educational. It makes you feel like you can talk about what happened and what you can do differently next time’^15^.

### Recommended changes to practice

All articles discussed how health professionals could be better supported following a surgical incident. A list of potential recommendations is shown in *Table* 1.

**Table 1:** Potential recommendations from selected articles

Recommendations	Brief description from articles
One‐to‐one support sessions	An informal one‐to‐one discussion with a senior colleague soon after the incident, with a second follow‐up meeting if necessary^4^ ^7^, ^8^, ^9^ ^15^, ^16^ ^18^, ^19^ ^26^, ^27^ ^30^, ^31^, ^32^, ^33^
Debriefing sessions	Debriefing sessions to help deconstruct the incident and encourage learning^7^ ^25^, ^29^ ^33^
	Trained psychologists would carry out formal debriefing sessions with the individual, similar to those carried out in the aviation industry^4^ ^7^
Mentoring	Putting structured peer support or mentoring programmes in place where the affected health professionals would be followed up by a senior colleague or manager soon after an event^7^ ^19^
Morbidity and mortality conferences (UK) Deaths and complications conferences (USA)	Morbidity and mortality, and deaths and complications conferences to be more structured and blame‐free, to encourage open discussions about an incident and promote a culture of shared learning within the organizations^7^ ^15^, ^26^ ^28^

	Opportunities to discuss freely an incident that they were involved in and draw on the experiences of senior colleagues across various specialties to promote learning^7^ ^15^, ^26^ ^28^
Education and training	Health professionals should be educated as part of their undergraduate curriculum about the possibility of surgical errors occurring in practice and what different coping strategies could be employed following these incidents^7^ ^15^, ^17^ ^26^, ^30^
Supportive environment	Organizations should promote an environment where mistakes from juniors are not perceived as their individual problems, but rather common glitches expected from trainees^26^
	The option to have some time off work in the aftermath of a surgical incident, as the psychological impact might affect their concentration and continued performance^7^ ^25^, ^29^ ^33^
	Managers and peers need to find time to listen and support the affected individuals. This support should be offered at an early stage following the event^7^ ^14^, ^21^
	Support systems should be structured and meet individual needs^21^
Investigation or inquiry process	To have an open and transparent process in analysing these events^19^
	A need for the formal investigation process to be explained more clearly following a surgical complication^19^ ^26^

Offering one‐to‐one support sessions to those affected by a surgical incident was viewed as particularly critical^4^ ^7^, ^8^, ^9^ ^15^, ^16^ ^18^, ^19^ ^27^, ^28^ ^32^, ^33^, ^34^, ^35^. This could take the form of an informal one‐to‐one discussion with a senior colleague soon after the incident, with a second follow‐up meeting if necessary. One UK general surgeon highlighted the importance of having someone more senior to speak to following the incident: ‘… it's very good to have someone a little more senior that if you have a problem you can say, ‘What am I going to do?’ or ‘What happens next?’ That's very, very unofficial’^7^. Some studies^5^ ^7^, ^8^ ^15^, ^19^ ^27^, ^34^ proposed that clinicians from various specialties be trained to support staff involved in an incident. One Canadian surgical trainee made comparisons with other industries, such as aviation, and how trained psychologists would carry out formal debriefing sessions with individuals to help them ‘figure out what went wrong, what was random’^27^. One anaesthetist was frustrated with the lack of organizational support offered to him: ‘[*NO ONE*] … sought to ask how I felt about it (patient death) and how it was affecting me’^31^.

A number of papers discussed the culture of surgery and the need for organizations to promote an environment where mistakes ‘are not viewed as problems with someone's character. Mistakes happen because you're a doctor in training and everyone has made a mistake at some point’^27^. Allowing staff to take time off after an incident was seen as an important element of support to the individual member of staff and protection for patients^31^. This might be a short period to enable the individual to reflect on the incident, although this was considered likely to depend on the individual^7^ ^15^, ^27^.

## Discussion

Health professionals can suffer severe emotional distress following a surgical incident. These incidents may arise from an operation that had a poor outcome, that might reflect the severity of illness of the patient, or an error assumed to be due to the health professional concerned or a member of the wider surgical team. This distress is influenced further by a number of other factors including the severity of the error, the individual's personality and character, and what, if any, support was offered by the individual's organization. This review has highlighted how health professionals viewed and reacted to these events, leading to a variety of coping strategies to regain their self‐confidence and positive thinking. It also identified the need for the development of an open culture of shared learning within an organization.

Most studies focused on the impact of surgical incidents on surgeons and anaesthetists, and neglected other members of the operating theatre team. Theatre nurses, other healthcare professionals and support workers can all experience emotional and psychological distress when involved in surgical incidents, with significant impact on their professional work^5^ ^8^, ^10^ ^36^. More research is needed to understand the impact of surgical incidents on the wider operating team.

This review also highlighted how a surgeon may become more risk‐averse following a surgical incident^7^. It is possible that some health professionals may subsequently be reluctant to perform a surgical procedure similar to that related to the incident. Similarly, other professionals who were part of the team that witnessed the incident may feel reluctant to work with others or participate in a similar procedure. Conversely, some staff seem more resilient following an event, reflecting and learning from the incident, and wishing to perform the same invasive procedure or get involved within the same environment to improve their self‐belief and confidence. There is a need to explore further attitudinal and behavioural changes towards patient safety following an event, and what knock‐on effects such decisions may have for patient care.

The majority of operating theatre staff felt that they received inadequate support from managers and peers within their organization following a surgical incident. Some staff were reluctant to discuss incidents for fear of retribution. This was an important finding, and highlighted the need for attitudinal change with respect to patient safety. Organizations need to cultivate a supportive environment to learn from incidents. Similar to the way in which the operating team comes together to complete the WHO Surgical Safety Checklist for every patient undergoing a surgical procedure, they could also collectively reflect on surgical incidents that occurred and identify learning points. One suggestion might be to draw on insights from the aviation industry, where trained psychologists carry out formal debriefing sessions with individuals and teams to help them ‘figure out what went wrong’^37^. The aviation industry places more emphasis on structured systems that link the adverse event to learning from it. Tools should be developed to help the team deconstruct surgical incidents that occur^38^.

This review also highlighted other ways that health professionals could be better supported following a surgical incident. Individuals need to be able informally to discuss the incident with a senior colleague or mentor soon after it has occurred^7^ ^19^. This would give them the opportunity to reflect with another experienced healthcare professional on what happened, possibly drawing on their knowledge or experience to promote learning and rebuild the individual's self‐confidence. Morbidity and mortality meetings need to be structured and blame‐free to encourage open discussions about an incident and promote a culture of shared learning within organizations^7^ ^15^, ^27^ ^29^. Organizations themselves need to cultivate a culture of ‘psychological safety’, whereby any member of staff can ask questions and receive feedback without appearing incompetent, so that new ways of working can be considered. This culture of psychological safety could potentially reduce the impact of incidents on individuals and promote learning.

Those responsible for the provision and organization of surgical services must also recognize the need not only to work with frontline staff to learn from these incidents, but also to disseminate lessons learned across their organizations effectively.

This review has limitations. Most of the included studies were conducted in North America. The review did not include studies that focused solely on the effect of malpractice claims on health professionals following an adverse incident. Although outside the scope of this review, these studies may have provided further insight into the emotional effects of incidents on theatre staff and their long‐term consequences. Furthermore, as part of the quality assessment of articles, more than half of the included articles used only one method to obtain data, which could be viewed as a weakness in these particular studies.

**Supporting information**

Additional supporting information may be found online in the supporting information tab for this article.

## Supporting information


**Appendix S1.** List of MeSH terms and text words used in databases: MEDLINE, EMBASE, CINAHL and PsycINFO
**Table S1** Key characteristics and findings of selected articles
**Table S2** Subthemes and overarching themes extracted from each article in the review

## References

[1] Classen DC , Resar R , Griffin F , Federico F , Frankel T , Kimmel N *et al* ‘Global trigger tool’ shows that adverse events in hospitals may be ten times greater than previously measured. Health Aff (Millwood) 2011; 30: 581–589. 21471476 10.1377/hlthaff.2011.0190

[2] Vincent C , Neale G , Woloshynowych M . Adverse events in British hospitals: preliminary retrospective record review. BMJ 2001; 322: 517–519. 11230064 10.1136/bmj.322.7285.517 PMC26554

[3] NHS Improvement . *Provisional Publication of Never Events Reported as Occurring Between 1 April 2016 and 31 March 2017*; 2017 https://improvement.nhs.uk/uploads/documents/Provisional_Never_Events_April_2016_-_March_2017.pdf [accessed 1 June 2017].

[4] Pratt S , Kenney L , Scott SD , Wu AW . How to develop a second victim support program: a toolkit for health care organizations. Jt Comm J Qual Patient Saf 2012; 38: 235–240. 22649864 10.1016/s1553-7250(12)38030-6

[5] Seys D , Wu AW , Van Gerven E , Vleugels A , Euwema M , Panella M *et al* Health care professionals as second victims after adverse events: a systematic review. Eval Health Prof 2013; 36: 135–162. 22976126 10.1177/0163278712458918

[6] Scott SD , Hirschinger LE , Cox KR , McCoig M , Hahn‐Cover K , Epperly KM *et al* Caring for our own: deploying a systemwide second victim rapid response team. Jt Comm J Qual Patient Saf 2010; 36: 233–240. 20480757 10.1016/s1553-7250(10)36038-7

[7] Pinto A , Faiz O , Bicknell C , Vincent C . Surgical complications and their implications for surgeons' well‐being. Br J Surg 2013; 100: 1748–1755. 24227360 10.1002/bjs.9308

[8] Sirriyeh R , Lawton R , Gardner P , Armitage G . Coping with medical error: a systematic review of papers to assess the effects of involvement in medical errors on healthcare professionals' psychological well‐being. Qual Saf Health Care 2010; 19: e43. 10.1136/qshc.2009.035253 20513788

[9] Pinto A , Faiz O , Bicknell C , Vincent C . Acute traumatic stress among surgeons after major surgical complications. Am J Surg 2014; 208: 642–647. 25241953 10.1016/j.amjsurg.2014.06.018

[10] Chard R . How perioperative nurses define, attribute causes of, and react to intraoperative nursing errors. AORN J 2009; 91: 132–145. 10.1016/j.aorn.2009.06.028 20102810

[11] Care Quality Commission . *Learning from Serious Incidents in NHS Acute Hospitals: a Review of the Quality of Investigation Reports*; 2016. http://www.cqc.org.uk/content/briefing-learning-serious-incidents-nhs-acute-hospitals [accessed 25 February 2017].

[12] NHS England . *NHS Staff Health and Wellbeing: CQUIN Supplementary Guidance*; 2016 https://www.england.nhs.uk/wp-content/uploads/2016/03/HWB-CQUIN-Guidance.pdf [accessed 28 May 2017].

[13] Aasland OG , Førde R . Impact of feeling responsible for adverse events on doctors' personal and professional lives: the importance of being open to criticism from colleagues. Qual Saf Health Care 2005; 14: 13–17. 15691998 10.1136/qshc.2002.003657 PMC1743972

[14] Edrees HH , Paine LA , Feroli ER , Wu AW. Health care workers as second victims of medical errors. Pol Arch Med Wewn 2011; 121: 101–107. 21532531

[15] Engel KG , Rosenthal M , Sutcliffe KM . Residents' responses to medical error: coping, learning, and change. Acad Med 2006; 81: 86–93. 16377827 10.1097/00001888-200601000-00021

[16] Harrison R , Lawton R , Perlo J , Gardner P , Armitage G , Shapiro J . Emotion and coping in the aftermath of medical error: a cross‐country exploration. J Patient Saf 2015; 11: 28–35. 25695552 10.1097/PTS.0b013e3182979b6f

[17] Hu YY , Fix ML , Hevelone ND , Lipsitz SR , Greenberg CC , Weissman JS *et al* Physicians' needs in coping with emotional stressors: the case for peer support. Arch Surg 2012; 147: 212–217. 22106247 10.1001/archsurg.2011.312 PMC3309062

[18] Mira JJ , Carrillo I , Lorenzo S , Ferrús L , Silvestre C , Pérez‐Pérez P *et al*; Research Group on Second and Third Victims. The aftermath of adverse events in Spanish primary care and hospital health professionals. BMC Health Serv Res 2015; 15: 151. 25886369 10.1186/s12913-015-0790-7 PMC4394595

[19] Ullström S , Sachs MA , Hansson J , Øvretveit J , Brommels M . Suffering in silence: a qualitative study of second victims of adverse events. BMJ Qual Saf 2014; 23: 325–332. 10.1136/bmjqs-2013-002035 PMC3963543 24239992

[20] Vinson AE , Mitchell JD . Assessing levels of support for residents following adverse outcomes: a national survey of anesthesia residency programs in the United States. Med Teach 2014; 36: 858–866. 24804917 10.3109/0142159X.2014.910299

[21] Seys D , Scott S , Wu A , Van Gerven E , Vleugels A , Euwema M *et al* Supporting involved health care professionals (second victims) following an adverse health event: a literature review. Int J Nurs Stud 2013; 50: 678–687. 22841561 10.1016/j.ijnurstu.2012.07.006

[22] Moher D , Shamseer L , Clarke M , Ghersi D , Liberati A , Petticrew M *et al*; PRISMA Group. Preferred Reporting Items for Systematic Review and Meta‐Analysis Protocols (PRISMA‐P) 2015 statement. Syst Rev 2015; 4: 1. 25554246 10.1186/2046-4053-4-1 PMC4320440

[23] Popay J , Roberts H , Sowden A , Petticrew M , Arai L , Rodgers M *et al* Guidance on the Conduct of Narrative Synthesis in Systematic Reviews: A Product from the ESRC Methods Programme. Version 1. Lancaster University: Lancaster, 2006.

[24] Gale N , Heath G , Cameron E , Rashid S , Redwood S . Using the framework method for the analysis of qualitative data in multi‐disciplinary health research. BMC Med Res Methodol 2013; 13: 117. 24047204 10.1186/1471-2288-13-117 PMC3848812

[25] Critical Appraisal Skills Programme (CASP) . *10 Questions to Help You Make Sense of Qualitative Research*; 2013. http://media.wix.com/ugd/dded87_29c5b002d99342f788c6ac670e49f274.pdf [accessed 18 December 2016].

[26] Amato PE , Malik ZM , Durieux ME , Gazoni FM . Emotional impact of perioperative catastrophes on anesthesiologists. Anesth Analg 2010; 110(Suppl 1): S214. 10.1213/ANE.0b013e318227524e 21737706

[27] Balogun JA , Bramall AN , Bernstein M . How surgical trainees handle catastrophic errors: a qualitative study. J Surg Educ 2015; 72: 1179–1184. 26073715 10.1016/j.jsurg.2015.05.003

[28] Luu S , Patel P , St‐Martin L , Leung SOS , Regehr G , Murnaghan ML *et al* Waking up the next morning: surgeons' emotional reactions to adverse events. Med Educ 2012; 46: 1179–1189. 23171260 10.1111/medu.12058

[29] Skevington SM , Langdon JE , Giddins G. ‘Skating on thin ice?’ Consultant surgeon's contemporary experience of adverse surgical events. Psychol Health Med 2012; 17: 1–16. 22191491 10.1080/13548506.2011.592841

[30] Creswell JW . Educational Research: Planning, Conducting, and Evaluating Quantitative and Qualitative Research (5th edn). Pearson Education: Upper Saddle River, 2014.

[31] Heard GC , Thomas RD , Sanderson PM . In the aftermath: attitudes of anesthesiologists to supportive strategies after an unexpected intraoperative patient death. Anesth Analg 2016; 122: 1614–1624. 27101503 10.1213/ANE.0000000000001227

[32] Bognar A , Barach P , Johnson JK , Duncan RC , Birnbach D , Woods D *et al* Errors and the burden of errors: attitudes, perceptions, and the culture of safety in pediatric cardiac surgical teams. Ann Thorac Surg 2008; 85: 1374–1381. 18355531 10.1016/j.athoracsur.2007.11.024

[33] Waterman AD , Garbutt J , Hazel E , Dunagan WC , Levinson W , Fraser VJ *et al* The emotional impact of medical errors on practicing physicians in the United States and Canada. Jt Comm J Qual Patient Saf 2007; 33: 467–476. 17724943 10.1016/s1553-7250(07)33050-x

[34] Patel AM , Ingalls NK , Mansour MA , Sherman S , Davis AT , Chung MH . Collateral damage: the effect of patient complications on the surgeon's psyche. Surgery 2010; 148: 824–830. 20727563 10.1016/j.surg.2010.07.024

[35] Gorini A , Miglioretti M , Pravettoni G . A new perspective on blame culture: an experimental study. J Eval Clin Pract 2012; 18: 671–675. 22435605 10.1111/j.1365-2753.2012.01831.x

[36] Copping C . Preventing and reporting drug administration errors. Nurs Times 2005; 33: 32–34. 16130498

[37] Kapur N , Parand A , Soukup T , Reader T , Sevdalis N . Aviation and healthcare: a comparative review with implications for patient safety. JRSM Open 2016; 7: 2054270415616548. 10.1177/2054270415616548 PMC4710114 26770817

[38] University of Aberdeen . *The Non‐Technical Skills for Surgeons (NOTSS) System Handbook v1.2: Structuring Observation, Rating and Feedback of Surgeons' Behaviours in the Operating Theatre*; 2012. https://www.iscp.ac.uk/static/help/NOTSS_Handbook_2012.pdf [accessed 5 September 2017].

