# Supplementary material for: Systematic review of psychological, emotional and behavioural impacts of surgical incidents on operating theatre staff
Source: BJS Open. 2017 Oct 26;1(4):106–13. doi: 10.1002/bjs5.21 (PMC5989958; doi:10.1002/bjs5.21)
Supplement: Supplementary file 1 — Appendix S1. List of MeSH terms and text words used in databases: MEDLINE, EMBASE, CINAHL and PsycINFO Table S1 Key characteristics and findings of selected articles Table S2 Subthemes and overarching themes extracted from each article in the review [file BJS5-1-106-s001.docx]

**BJS5_21**

**Systematic review of psychological, emotional and behavioural impacts of surgical incidents on operating theatre staff**

**N. Serou, L. Sahota, A. K. Husband, S. P. Forrest, K. Moorthy, C. Vincent, R. D. Slight and S. P. Slight**

**Appendix S1** List of MeSH terms and text words used in databases: MEDLINE, EMBASE, CINAHL and PsycINFO

1. surg* error*.mp. [mp=title, abstract, original title, name of substance word, subject heading word, keyword heading word, protocol supplementary concept word, rare disease supplementary concept word, unique identifier]

2. Medical Errors/

3. surg* mistake*.mp. [mp=title, abstract, original title, name of substance word, subject heading word, keyword heading word, protocol supplementary concept word, rare disease supplementary concept word, unique identifier]

4. surg* fault*.mp. [mp=title, abstract, original title, name of substance word, subject heading word, keyword heading word, protocol supplementary concept word, rare disease supplementary concept word, unique identifier]

5. surg* failure*.mp. [mp=title, abstract, original title, name of substance word, subject heading word, keyword heading word, protocol supplementary concept word, rare disease supplementary concept word, unique identifier]

6. adverse event*.mp. [mp=title, abstract, original title, name of substance word, subject heading word, keyword heading word, protocol supplementary concept word, rare disease supplementary concept word, unique identifier]

7. never event*.mp. [mp=title, abstract, original title, name of substance word, subject heading word, keyword heading word, protocol supplementary concept word, rare disease supplementary concept word, unique identifier]

8. operat* error*.mp. [mp=title, abstract, original title, name of substance word, subject heading word, keyword heading word, protocol supplementary concept word, rare disease supplementary concept word, unique identifier]

9. surg* complication*.mp. [mp=title, abstract, original title, name of substance word, subject heading word, keyword heading word, protocol supplementary concept word, rare disease supplementary concept word, unique identifier]

10. catastrophic error*.mp. [mp=title, abstract, original title, name of substance word, subject heading word, keyword heading word, protocol supplementary concept word, rare disease supplementary concept word, unique identifier]

11. surg* pitfall*.mp. [mp=title, abstract, original title, name of substance word, subject heading word, keyword heading word, protocol supplementary concept word, rare disease supplementary concept word, unique identifier]

12. surg* incident*.mp. [mp=title, abstract, original title, name of substance word, subject heading word, keyword heading word, protocol supplementary concept word, rare disease supplementary concept word, unique identifier]

13. surg* event*.mp. [mp=title, abstract, original title, name of substance word, subject heading word, keyword heading word, protocol supplementary concept word, rare disease supplementary concept word, unique identifier]

14. (wrong* adj2 surg*).mp. [mp=title, abstract, original title, name of substance word, subject heading word, keyword heading word, protocol supplementary concept word, rare disease supplementary concept word, unique identifier]

15. (surg* adj2 error*).mp. [mp=title, abstract, original title, name of substance word, subject heading word, keyword heading word, protocol supplementary concept word, rare disease supplementary concept word, unique identifier]

16. (surg* adj2 event*).mp. [mp=title, abstract, original title, name of substance word, subject heading word, keyword heading word, protocol supplementary concept word, rare disease supplementary concept word, unique identifier]

17. (surg* adj2 incident*).mp. [mp=title, abstract, original title, name of substance word, subject heading word, keyword heading word, protocol supplementary concept word, rare disease supplementary concept word, unique identifier]

18. (surg* adj2 complication*).mp. [mp=title, abstract, original title, name of substance word, subject heading word, keyword heading word, protocol supplementary concept word, rare disease supplementary concept word, unique identifier]

19. exp Surgeons/

20. surgeon*.mp. [mp=title, abstract, original title, name of substance word, subject heading word, keyword heading word, protocol supplementary concept word, rare disease supplementary concept word, unique identifier]

21. physicians/ or exp surgeons/

22. surg* trainee*.mp. [mp=title, abstract, original title, name of substance word, subject heading word, keyword heading word, protocol supplementary concept word, rare disease supplementary concept word, unique identifier]

23. resident*.mp. [mp=title, abstract, original title, name of substance word, subject heading word, keyword heading word, protocol supplementary concept word, rare disease supplementary concept word, unique identifier]

24. an*sthetist*.mp. [mp=title, abstract, original title, name of substance word, subject heading word, keyword heading word, protocol supplementary concept word, rare disease supplementary concept word, unique identifier]

25. operating personnel.mp. [mp=title, abstract, original title, name of substance word, subject heading word, keyword heading word, protocol supplementary concept word, rare disease supplementary concept word, unique identifier]

26. operating staff*.mp. [mp=title, abstract, original title, name of substance word, subject heading word, keyword heading word, protocol supplementary concept word, rare disease supplementary concept word, unique identifier]

27. theatre staff*.mp. [mp=title, abstract, original title, name of substance word, subject heading word, keyword heading word, protocol supplementary concept word, rare disease supplementary concept word, unique identifier]

28. exp Operating Room Technicians/

29. Operating department practitioner*.mp.

30. theatre nurse*.mp. [mp=title, abstract, original title, name of substance word, subject heading word, keyword heading word, protocol supplementary concept word, rare disease supplementary concept word, unique identifier]

31. health professional*.mp. [mp=title, abstract, original title, name of substance word, subject heading word, keyword heading word, protocol supplementary concept word, rare disease supplementary concept word, unique identifier]

32. health personnel/ or faculty, nursing/ or nurse anesthetists/ or exp nurse clinicians/

33. exp Emotions/

34. attitude/ or exp "attitude of health personnel"/

35. personal qualit*.mp. [mp=title, abstract, original title, name of substance word, subject heading word, keyword heading word, protocol supplementary concept word, rare disease supplementary concept word, unique identifier]

36. personal attribute*.mp. [mp=title, abstract, original title, name of substance word, subject heading word, keyword heading word, protocol supplementary concept word, rare disease supplementary concept word, unique identifier]

37. personal perception*.mp. [mp=title, abstract, original title, name of substance word, subject heading word, keyword heading word, protocol supplementary concept word, rare disease supplementary concept word, unique identifier]

38. professionalism.mp. [mp=title, abstract, original title, name of substance word, subject heading word, keyword heading word, protocol supplementary concept word, rare disease supplementary concept word, unique identifier]

39. exp Professionalism/

40. exp Stress, Psychological/

41. psychosocial.mp. [mp=title, abstract, original title, name of substance word, subject heading word, keyword heading word, protocol supplementary concept word, rare disease supplementary concept word, unique identifier]

42. Behavior/

43. behav*r*.mp. [mp=title, abstract, original title, name of substance word, subject heading word, keyword heading word, protocol supplementary concept word, rare disease supplementary concept word, unique identifier]

44. exp Psychosocial Deprivation/

45. 1 or 2 or 3 or 4 or 5 or 6 or 7 or 8 or 9 or 10 or 11 or 12 or 13 or 14 or 15 or 16 or 17 or 18

46. 19 or 20 or 21 or 22 or 23 or 24 or 25 or 26 or 27 or 28 or 29 or 30 or 31 or 32

47. 33 or 34 or 35 or 36 or 37 or 38 or 39 or 40 or 41 or 42 or 43 or 44

48. 45 and 46 and 47

**Table S1** Key characteristics and findings of selected articles

| **Author and Time of study** | **Country, Sample and Setting** | **Design or Methods used** | **Type of errors** | **Aims and Objectives** | **Outcomes/recommendations** | **Quality Appraisal Score/CASP score/Triangulation (Yes/No)** |
| --- | --- | --- | --- | --- | --- | --- |
| Aasland, O. G. and R. Forde (2005). | **Location:**  Norway  **Sample size:**  1318 doctors with various specialities | Quantitative methodology: postal questionnaires | Surgical and all other type of errors | To explore the responsibility felt by the senior and junior doctors upon adverse incidents and its impact on their life and their experience in accepting criticisms aftermath. | Study shows that severe patient injury is more prevalent in doctors working in surgical specialities and it has a negative impact on their personal and professional lives.  **Recommendations:**  Study recommended to have a work environment where shared and communal criticism with constructive feedback is encouraged, which results in reduced negative impact on doctors following an event. | **Triangulation:** No |
| Amato, P. E., et al. (2010) | **Location:** USA,  **Sample size :**  659 Anaesthetists  12 volunteers for telephone interviews | Quantitative and qualitative methodology: postal survey and semi-structured telephone interviews | Perioperative errors | Emotional impact on anaesthetists upon perioperative catastrophic errors and their long term ability to provide care to patients in operating room | Study indicated that upon experiencing a catastrophic error, most of the respondents experienced a high degree of emotional impact with a majority of respondents experiencing guilt, depression, anxiety, sleeplessness, fear of litigation, fear of judgment by colleagues and anger.  **Recommendations**  Recommended to design and process protocols and standard procedures explaining the support and guidance required for the practitioners upon experiencing a catastrophic error and a need to embed training programmes for all medical practitioners in handling adverse events to not only to protect the well-being of anaesthetists but also helps to prevent harm to patients. | **CASP score**- 8/10  **Triangulation**-Yes |
| Balogun, J. A., et al. (2015). | **Location:** Tertiary care hospital in Toronto, Canada  **Sample size:** 23 surgery residents: Neurosurgical, general, Vascular and ENT specialities. | A Qualitative Study | Surgical Catastrophic errors | To explore various coping mechanisms of surgical trainees upon experienced with catastrophic surgical errors  To recommend support strategies or schemes for surgeons affected by surgical errors. | The study found that most errors are due to system failures. Surgical trainees where not trained in how to cope with medical errors. There is lack of communication relationships between senior surgeons and surgical trainees to discuss about their medical errors. Some trainees use surgical complications as learning experiences for future practices and most of them felt that seeking emotional support is always perceived as personal weakness and it underlines the attitude of the surgical profession.  **Recommendations:** Some surgical trainees upon impact with an error might need counselling or debriefing sessions to support them. At the beginning of their surgical careers they need to be taught in how to cope with medical errors and senior surgeons need to fill the communication gap between themselves and their juniors and discuss their experiences and provide unconditional support when trainees are experienced with catastrophic surgical errors. | **CASP score:** 9/10  **Triangulation:** No |
| Bognar, A., et al. (2008) | **Location:** Three academic teaching hospitals, Boston, USA  Paediatric Cardiac Surgical team members.  **Sample size:** 61  24:anaesthetists, 15 :nurses or technicians (scrub, circulating, and one physician assistant)  10: perfusionists, 7 surgeons, and 5 participants did not indicate their profession. | Quantitative Methodology. Surveys, Questionnaires involving open ended questions, Scaled questions and questions regarding Clinical scenarios of Adverse Event | Clinical errors in Perioperative Care-Surgical Errors | To explore the impact of real and prospective surgical errors on Paediatric surgical teams (PCS) and its effect on their performance individually and as a team.  To explore their attitude and perception of safety culture. | Most of the participants feel burden when errors occur.  Only a small percentage of participants reported that debriefing occurs after an error has occurred and resulted in patient harm.  **Recommendations:**  Team members to be given responsibility and power to address safety issues to the health management in order to reduce errors and patient harm. Briefing and debriefing need to be done before and after an error has occurred. | **Triangulation:** No |
| Chard R(2010) | **Location:** USA  **Sample Size:** 272 Perioperative registered nurses | Quantitative methodology, Questionnaires | Intraoperative errors, surgical errors | -To investigate the definitions, conditions, and perceived  causes of intraoperative nursing errors  -Impact of Intraoperative errors on perioperative nurses, and  examine coping strategies | Inexperience, lack of supervision, work overload, and faulty judgement are the factors found to be causes of intraoperative errors.  Perioperative nurses experienced emotional distress, angry to themselves, angry at others, and embarrassed following an error in theatre  **Recommendations:** Need for qualitative study to explore perioperative nurses’ personal experiences following an error and the need for theatre nurses to understand the meaning of “error” to report. | **Triangulation:** No |
| Engel, K. G., et al. (2006). | **Location:** USA, 600-bed teaching hospital.  **Sample size**:26 residents:  5-Surgery  17-Medicine  4-Obs/Gynaecology | Qualitative methodology with semi-structured interviews | All types of errors include Surgical errors | -To examine the challenges facing physicians upon experienced with medical errors and their perception to medical errors  - To explore their coping strategies once faced with these medical errors | Study shows the residents upon experiencing a medical error, feel emotionally distress, guilt, fear, anger and isolated  Residents cope with these experiences by talking to medical professionals, friends, family and patients itself.  Few residents cope by getting involved in physical activities such as in sports.  **Recommendations:**  Need for education and training to educate residents to cope after been affected by medical errors,  Need for more formal and informal conference forums to discuss the errors with fellow experienced colleagues  Need for more structured programmes to provide emotional support to residents after medical or surgical complications. | **CASP score**-9/10  **Triangulation**- No |
| Harrison, R., et al. (2015). | **Location:** two large teaching hospitals in UK and USA  **Sample size**: 265 physicians and Nurses: 120 physicians and 145 nurses (total 265).  UK sample 61 physicians and 65 nurses (total 126), and the U.S. sample 59 physicians and 80 nurses (total 139). Senior physicians included consultant physicians (50); junior physicians included house officers, senior house officers, registrars, interns, residents, and fellows (70); senior nurses included band 7 and 8 nurses and nurse specialists (49); and junior nurses were band 5 and 6 or registered nurses (96). | Quantitative methodology, Cross-sectional and cross country survey | All type of medical errors including surgical errors | To investigate professional and personal impact on physicians and nurses after an error.  To explore various emotional responses based on type of error, location and copying strategies shown by health professionals, whether they are different based on location?  Awareness of supporting systems by professionals and their willingness to seek support from organisation | UK professionals experience more physical and personal interference than professionals in USA after involving in an error. Nurses tend have more negative impact emotionally than physicians from both countries.  Most of the professionals from both countries were not aware of the supporting services available in their organisation and they are willing to use the services if the support is given by their peers or seniors in a supportive and confidential environment. **Recommendations:** Health organisations need to develop a clinical support programme which is more structured and encourages supporting the affected professionals in a supportive environment which enables the professionals to have an open and honest discussion about their errors. This will help in increase in staff morale, error reporting and opportunities to learn and develop. | **Triangulation:** No |
| Heard, G. C., et al. (2016) | **Location**: Australia.  **Sample size:**  766 anaesthetists of varied experiences. | Quantitative methodology, postal questionnaires | Adverse incidents in operating theatres involving anaesthetists | To study the support required for the anaesthetists when there is a catastrophic death in operating theatre.  Compare the support required when the death occurs due to an anaesthetist error or without. | The study shows that the anaesthetists do need emotional support when there is catastrophic death which might involve error or not. They need peers support and counselling guidance when affected by it.  The respondents in the study who are affected with a death in theatre due to their error recommended to have a day off work from duty, out of hours counselling services when needed, peers support and professional advice | **Triangulation:** No |
| Hu, Y. Y., et al. (2012) | **Location:** A large tertiary care academic hospital  **Sample size:** 108 residents and attending physicians in the department of surgery, emergency medicine and anaesthesia | Quantitative methodology include survey questionnaires | Medical errors including surgical errors | To explore the physicians needs in coping with the emotional stressors (include medical errors)  To examine the willingness of physicians to seek institutional support designed for physicians who in distress | The study shows that most of the respondents seek support when affected with adverse events when compared to other distress caused during every day work such as complaints or conflicts at work place.  Lack of time and fear of confidentiality breach is a barrier for the professionals in not seeking help when affected with medical errors.  Anaesthetists and surgeons are more likely to seek support when affected with adverse events when compared to residents from emergency medicine.  Most of the physicians and residents are not aware of the support services available in their organisations and to the health professions in general when affected with an medical error or any other distress caused in the work place | **Triangulation:** No |
| Luu, S., et al. (2012) | **Location:** three academic hospitals in large urban setting in Canada.  **Sample:**20 surgeons  13 general surgeons, 3 neurosurgeons, one cardiac surgeon, one urology surgeon, one gynaecology surgeon, one vascular surgeon | Qualitative methodology. Grounded Theory. Semi-structured and structured interviews. | Surgical errors | To explore surgeons’ response and reactions to surgical errors.  Their effect on further surgeons clinical decision making | The study shows that all surgeons have shown four phases of reactions when affected by adverse events.  Kick: It is the initial phase where there is physiological and emotional effect  Fall: Soon after the initial phase the extent of error and their contribution the error will be analysed  Recovery: This period is where the surgeons reflect on the error and pick up learning points  Long-term impact: is when the surgeons experience the impact of error throughout their profession based on individual characteristics and personalities.  Need for more support on individual basis to surgeons affected by errors or adverse events to improve their well-being and future clinical decision making. | **CASP**:9/10  **Triangulation:** Yes |
| M. Skevington, S., et al. (2012). | **Location:** Royal United Hospital, UK  **Sample size:**  11 consultant surgeons with varied speciality:  orthopaedics, obstetrics,  otolaryngology, urology, vascular and general surgery. | Qualitative methodology , Semi structured interviews | Surgical errors | The aim of the study is to investigate senior consultant surgeons perspective and views of adverse surgical events (ASE) | The study highlighted the environmental, organisation and social factors contributing to the adverse surgical events (ASE).  Strategies in dealing with ASE are diverse and pragmatic.  **Recommendations:**  Good and self-confident leadership is needed at hierarchies to promote solutions during ASE’s within the organisation | **CASP:** 8/10  **Triangulation:** No |
| Mira, J. J., et al. (2015). | **Location:** 8 Primary care health centres and Hospitals in Spain.  **Sample**: 1087 health professionals Medical and Surgical , 610 from Primary care and  477 from hospitals. | Quantitative methodology, Cross-sectional survey | All type of medical errors include surgical errors | The aim of the study is to explore the effect of adverse events on Spain health professionals personally and professionally. | Study shows 6 out of 10 health professionals in Spain encounter adverse events either directly or indirectly. They experience fear, anxiety and guilt aftermath. Spain doctors worry more than nurses about the negative consequences after adverse events, professionally and personally.  Spain health professionals hardly receive any training and support when compared to other countries in coping with adverse events.  **Recommendations:** Institutional support mechanisms need to be enforced to support second victims, I, e Health professionals after an adverse incidents in Spain Hospitals. | **Triangulation:** No |
| Patel, A. M., et al. (2010) | **Location:** Michigan  State University, USA  **Sample Size:** 123 surgeons with varied speciality  General surgery 75/123  Trauma 40/123  Critical care 29/123  Vascular surgery 18/123  Orthopaedic surgery 15/123  Specialised breast surgery 13/123  Other surgical specialties-less than 10% | Quantitative methodology, Cross-sectional survey | Surgical errors | Aim of the study is to investigate the effect of surgical errors on surgeons emotionally and their effect on performance in clinical practice.  The study also aims to identify the coping mechanisms used by the surgeons once involved in a surgical adverse event. | The study highlights that surgeons have an emotional professional impact once experienced with first major patient complication in practice after leaving residency.  Same amount of support or guidance is lacking in practice compared to being in residency  **Recommendations:** efforts need to be made to make all clinicians aware of the supporting systems for surgeons and recognise unrecognised emotional effects following patients complication | **Triangulation:** No |
| Pinto, A., et al. 2013). | **Location:** 2 NHS teaching hospitals in London, UK  **Sample:** 27 surgeons: General and Vascular surgeons both consultant and vascular surgeons. | Qualitative methodology, Semi- structured interviews | Surgical errors | -The study aims to explore the effect of surgical errors on surgeons and their coping strategies and perception of support.  -Adverse incidents impact on surgeons well-being | The study shows that the surgeons are affected emotionally after a surgical complication. Immediate reactions include anger and anxiety about their career and the consequences of an error. Senior surgeons deal with complications effectively when compared to junior surgeons.  Peer support and relationship after surgical event is vital and the need for structured debriefings after the surgical event is important as well.  **Recommendations:** Mentoring is key to support surgeons after affected by surgical complication  Morbidity and Mortality meetings need to be reformed as educational forums  Teamwork in dealing with complex cases during critical clinical decision making and Psychological support including a structured clinical support programmes need to be developed to provide emotional support for surgeons affected with surgical complications | **CASP score:** 9/10  **Triangulation:** No |
| Pinto, A., et al. (2014). | **Location:** 3 NHS trusts in London, UK  **Sample:** 47 general and vascular surgeons | Quantitative methodology. Survey questionnaires | Surgical errors | To explore surgeons level of acute traumatic stress after surgical complications and their coping strategies.  To investigate surgeons perceptions of institutional safety culture on surgical complications | Surgeons do experience acute traumatic stress following a surgical complication.  Self-distraction is one of the coping strategies used by the surgeons.  **Recommendations:** Institutions to develop support training programmes to provide support to surgeons affected with surgical complication | **Triangulation:** No |
| Scot S D et al (2009) | **Location:** Columbia, USA  **Sample:**31 second victims.  10 physicians, 11 registered nurses, 10 other includes managers, therapists, physician assistants and scrub technicians | Qualitative methodology, Semi- structured interviews | All type of medical errors include surgical errors | To explore the recovery stages of second victims in health care after adverse patient events | Reported six stages of recovery  (1) chaos and accident response, (2)  intrusive reflections, (3) restoring personal integrity, (4) enduring  the inquisition, (5) obtaining emotional first aid and (6)  moving on. The sixth stage, moving on, led to one of three  outcomes: dropping out, surviving or thriving.  **Recommendations:**  The study recommended the need for trained supervisors and front line peers to provide and target support to the affected staff (second victims) at the early stages following an event rather than at later stages where the second victims can get support from other established support which most of the institutions acquire such as risk managers, palliative care practitioners, chaplains, social workers etc. | **CASP score:** 8/10  **Triangulation:** No |
| Ullström, S., et al. (2014). | **Location**: Swedish University Hospital, Sweden  **Sample:** 21 Health professionals.  Physicians:10  Nurses: 9  Allied health professionals:2 | Qualitative methodology, semi-structured interviews | All type of errors include surgical errors | To explore the effect of adverse incidents on Swedish health professionals and the support they require from the organisation and the support they receive. | The study shows the health professionals experience emotional distress. They are affected by the organisation reaction to the adverse event. There is lack of structure or support during formal investigative process; it is much unstructured and not systematic. The feedback on investigation is not given to the second victim on a timely manner.  **Recommendations:** Organisation and policy makers need to develop well structure supporting programmes for staff affected with adverse events and promote a culture of openness and transparency so that the affected second victims can be open and honest in discussing the errors to their peers and managers and thereby promote learning opportunities to develop | **CASP score:** 9/10  **Triangulation:** No |
| Vinson, A. E. and J. D. Mitchell (2014). | **Location:** American Colleges of Graduate Medical Education (ACGME), USA  **Sample:** 67 Programme director for anaesthesia residency programmes | Quantitative methodology, Survey. | Errors in operating theatres | The aim of the study is to determine the incidence, efficacy and utilisation of supporting programmes in place for the anaesthetic residents during their training when affected with adverse incidents | The study shows peer support and department meetings are extremely helpful for the residents. The morbidity and mortality forum conference forums after adverse incidents are useful. Residents utilise the department led supporting programmes and few residents are not aware of institutional supporting programmes when affected with adverse incidents | **Triangulation:** No |
| Waterman, A. D., et al. (2007). | **Location**: USA and Canada.  **Sample:** 3,171 of physicians :internal medicine, paediatrics, family medicine, and surgery | Quantitative methodology, cross-sectional survey. | All type of errors including surgical errors | Study aims to investigate the effect of medical errors on physicians job related stress and the support needed to cope with medical errors | Many physicians experience emotional distress following medical errors. They feel anxious about future errors due to lack of confidence, experience reduced job satisfaction and feel that the error and near misses has bought harm to their reputation.  **Recommendations:** Significant organisational support need to developed to guide physicians while experiencing with medical errors | **Triangulation:** No |

**Review Articles**

| **Author , Date** | **Types of articles included** | **Types of errors included** | **Aims and Objectives** | **Results and findings** |
| --- | --- | --- | --- | --- |
| Sirriyeh R et.al (2010) | Qualitative and Quantitative | All type of errors | Review of literature which include:   - Impact of a medical error on the health professional - Long term and short term coping methods after medical error - What factors influence the immediate   response to error and individual coping methods | - Psychological effect on affected individuals. - Increase assertiveness and improved peer relationships reported - Recommended for future research to explore the complete investigation of short term to long term impact of medical errors and the role of organisation and pre-reg education following errors. |
| Seys D et al (2013) | Qualitative and Quantitative | All type of errors | Review of literature on the care and support required to second victims at individual and organisational level | - Support systems need to be provided immediately following an error. - Support systems for the individuals need also to be concentrated at middle and long term basis following an error |

**Table S2** Subthemes and overarching themes extracted from each article in the review

| **Articles** | **Initial subthemes emerged** | **Overarching themes** |
| --- | --- | --- |
| Aasland, O. G. and R. Forde (2005). | Negative impact, Needed professional support, criticism and peer support | - The emotional impact on health professionals. - Organisation culture and support |
| Amato, P. E., et al. (2010) | Emotional and professional impact, peer and manager support, recommendations. | - The emotional impact on health professionals; - Organisation culture and support; - Recommended changes to practice. |
| Balogun, J. A., et al. (2015). | Errors due to system deficits, lessons learned from incidents, support for surgical trainees, counselling services to be offered, culture of surgery | - Learning from surgical complications - Organisation culture and support - Recommended changes to practice |
| Bognar, A., et al. (2008) | Work environment and attitudes lead to surgical incidents, psychological impact following an event , recommendations to improve communication and increase education and training, manager and peer support | - The emotional impact on health professionals - Organisation culture and support - Recommended changes to practice |
| Chard R(2010) | Emotional distress following an event Coping with intraoperative errors and relationship to change in practice, learning from perioperative errors | - The emotional impact on health professionals - Individual coping strategies - Learning from incidents |
| Engel, K. G., et al. (2006). | Emotional response, Coping mechanisms, learning and change of practice, recommendations | - The emotional impact on health professionals - Learning from Surgical Complications - Recommended changes to practice |
| Harrison, R., et al. (2015). | Personal and professional disruptions, Negative impact, emotional distress, inadequate organisation and peer support, problem focused coping methods | - The emotional impact on health professionals - Organisation culture and support - Individual coping strategies |
| Heard, G. C., et al. (2016) | Support from managers and peers after error, need for counselling , Emotional response | - The emotional impact on health professionals - Organisation culture and support |
| Hu, Y. Y., et al. (2012) | Physical or mental illness, lack of confidence, personal management after incident, recommendations | - The emotional impact on health professionals - Individual coping strategies - Recommended changes to practice |
| Luu, S., et al. (2012) | Emotional and professional impact, immediate and long term impact gain knowledge and expertise and learning from events. | - The emotional impact on health professionals - Individual coping strategies - Recommended changes to practice |
| M. Skevington, S., et al. (2012). | Negative emotions following an error, organisation and peer support needed, education and training , learning from errors | - The emotional impact on health professionals - Organisation culture and support - Learning from surgical complications |
| Mira, J. J., et al. (2015). | Varied emotional responses, organisational support , education and training needed for coping , recommendations | - The emotional impact on health professionals - Organisation culture and support - Individual coping strategies Learning from surgical complications |
| Patel, A. M., et al. (2010) | Emotional and Professional Impact, Need for support systems. | - The emotional impact on health professionals - Recommended changes to practice |
| Pinto, A., et al. (2013). | Emotional and professional impact, factors effecting surgical reactions after an error, Organisation culture, Coping methods, Learning and support following complications | - The emotional impact on health professionals - Organisation culture and support - Individual coping strategies. Learning from surgical complications. |
| Pinto, A., et al. (2014). | Post traumatic distress, organisation support and recommendations | - The emotional impact on health professionals - Organisation culture and support |
| Scot S D et al (2009) | Emotional and personal impact, coping and supporting systems-recommendations | - The emotional impact on health professionals - Recommended changes to practice |
| Ullström, S., et al. (2014). | Emotional distress, long term and short term impact, organisation support, recommendations | - The emotional impact on health professionals - Organisation culture and support - Recommended changes to practice |
| Vinson, A. E. and J. D. Mitchell (2014). | Recommendations and organisation support after an event | - Organisation culture and support - Recommended changes to practice |
| Waterman, A. D., et al. (2007). | Personal and professional impact, organisation support, recommendations | - The emotional impact on health professionals - Organisation culture and support - Recommended changes to practice |
| **Review Articles** | **Themes Emerged** |  |
| Sirriyeh R et .al (2010) | Response and impact, Coping and learning, Attitudes in the context of culture, Moderating factors and implications for practice, recommendations | - The emotional impact on health professionals - Organisation culture and support - Recommended Changes to practice |
| Seyes, D et. al (2013) | Support for second victims and recommendations | - Organisation culture and support - Recommended changes to practice |
